# Pass-Transistor-Enabled Split Input Voltage Level Shifter for Ultra-Low-Power Applications

**DOI:** 10.3390/mi16010064

**Published:** 2025-01-05

**Authors:** Chakali Chandrasekhar, Mohammed Mahaboob Basha, Sari Mohan Das, Oruganti Hemakesavulu, Mohan Dholvan, Javed Syed

**Affiliations:** 1 Department of ECE, Sri Venkateswara College of Engineering, Tirupati 517507, AP, India; dr.chandrasekhar.c@svcolleges.edu.in; 2 Department of ECE, Sreenidhi Institute of Science and Technology (Autonomous), Hyderabad 501301, TG, India; mohan.d@sreenidhi.edu.in; 3 Department of ECE, SVR Engineering College, Nandyal 518501, AP, India; mohandas.ece@svrec.ac.in; 4 Department of EEE, Annamacharya University, Rajampet 516126, AP, India; ohk@aitsrajampet.ac.in; 5 Department of Mechanical Engineering, College of Engineering, King Khalid University, Abha 61421, Saudi Arabia

**Keywords:** low threshold voltage, power-efficient, sub-threshold operation, multi-threshold CMOS, IoT applications

## Abstract

In modern ICs, sub-threshold voltage management plays a significant role due to its perspective on energy efficiency and speed performance. Level shifters (LSs) play a critical role in signal exchange among multiple voltage domains by ensuring signal integrity and the reliable operation of ICs. In this article, a Pass-Transistor-Enabled Split Input Voltage Level Shifter (PVLS) is designed for area, delay, and power-efficient applications with a wide voltage conversion range. The represented low-power LS structure is a general blend of both pull-up and pull-down networks that perform level-up or level-down shifts. The proposed PVLS is incorporated with the multi-threshold CMOS technique and a load-balancing driving split inverter to limit high static current, leakage power, and performance degradation. The schematic structure could be able to convert voltages from low to high as well as high to low. The architecture design has the lowest silicon area. The implementation of the proposed design was taken under 55 nm CMOS technology. The represented LS could be able to convert voltage ranges between 0.3 V and 1.3 V, which has a dynamic power of 2.00 nW. The overall propagation delay of the LS is 90 ps and an area of 7.66 µm^2^ for an input frequency of 1 MHz.

## 1. Introduction

**Figure 1:**
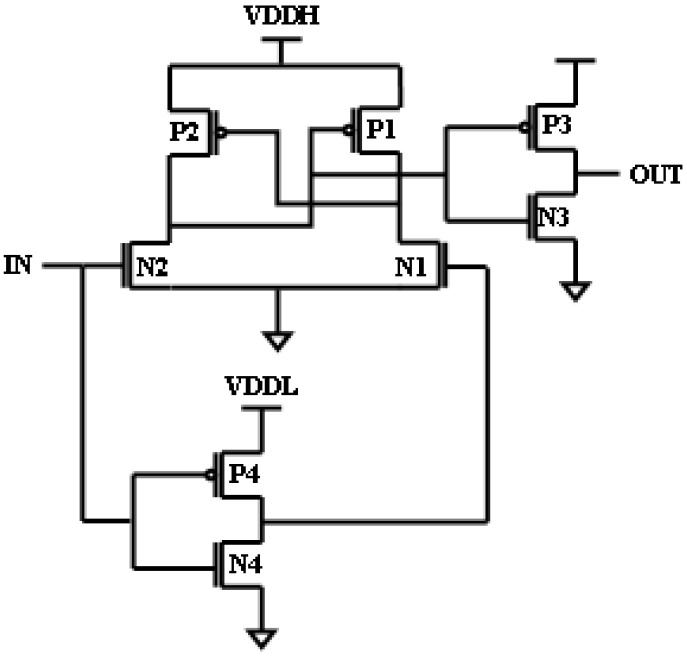
Conventional LS.

In the upward era of a digital world, the demand for low power from battery-operated devices such as wireless sensors, IoT devices, and mobile phones has been rising tremendously [1,2,3]. For the conservation of battery life span, there is a huge demand for low-power circuits. The digital systems functioning with diverse blocks with multiple supply voltages demand the voltage level shifting of the signals. This could be achieved with voltage LSs, which can voltage-shift low levels and deep sub-threshold-level voltages to high levels for the consequent block. The multiple supply voltages are used to maintain performance and power trade-off. MSVs are extensively used in systems on chips. In such systems on chips, LSs are needed to interconnect these logic devices across different supply voltages. Therefore, there might be a huge number of LSs in a block. So, there might be an increased delay, area, and power consumption [4,5,6]. In this article, power-efficient LS without sacrificing speed is designed, which also decreases the area and delay. Some of the core types of conventional LSs are considered and are described. Figure 1 shows the differential cascaded voltage switch (DCVS). The objective of this LS is to exchange the low-level voltage of input for a high-level output voltage. A strong contention can be observed among “pull-up and pull-down networks”. This tough contention can affect the voltage conversion in LSs. If the voltage of the lower supply of VDDL is lower than the threshold voltages of MOS transistors, then the pull-down network becomes weaker and can no longer withstand the pull-up network, so the voltage conversion fails. As the network becomes weaker, the flow of current through the pull-down network also diminishes. So, to intensify the current through the pull-down network, the firming of the pull-down network has to be achieved [7,8,9].

**Figure 2:**
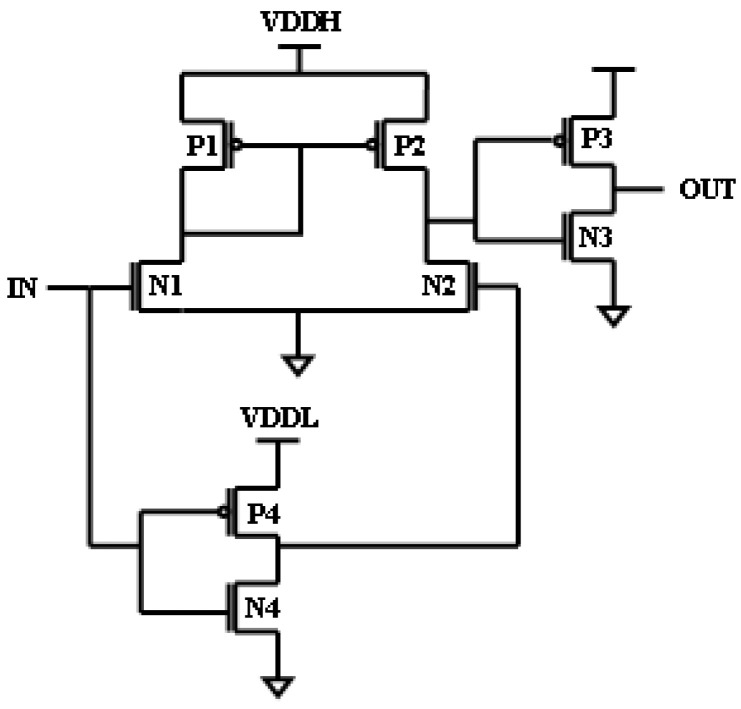
Wilson current mirror.

Figure 2 shows another conventional current mirror LS. This LS has the facility to shift exceedingly low-level voltage to high-level voltage, but the main disadvantage of this LS is power consumption. At a high input, the static current principles lead to an increase in power consumption. To rectify the drawbacks discussed above, some of the LSs were designed with current limiters to limit the power of the pull-up networks [10,11,12,13]. By limiting the force of the pull-up networks, we can eliminate the conflict between the “pull-up and pull-down networks”. But, using these limiters may result in huge power consumption, which is the drawback of this circuit.

**Figure 3:**
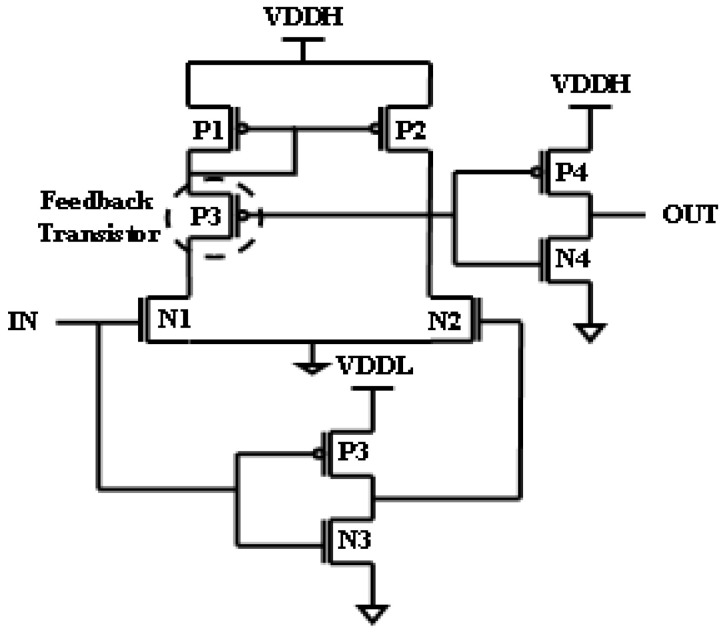
Feedback transistor LS.

**Figure 4:**
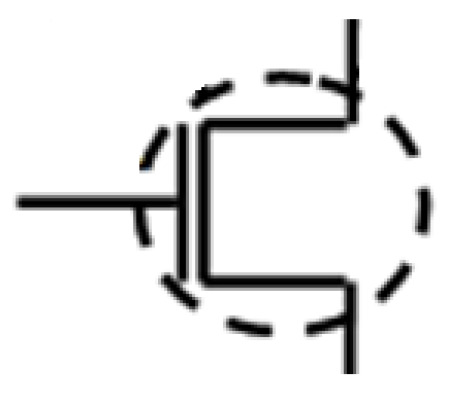
NMOS pass transistor.

Figure 3 demonstrates the LS established on Wilson’s current-mirror-based structure. The main objective of this LS is to limit the static current when the input is high, which is achieved by inserting a feedback transistor. But, these LSs have a drawback [14,15]. When the output is high, the static power increases at the output buffer due to the voltage drop. To eradicate these harms, a new LS has been designed, which is shown in Figure 4. Section 2 details the literature on LSs, Section 3 presents the proposed Pass-Transistor-Enabled Split Input Voltage Level Shifter, Section 4 deals with the results and a comparison of PVLS with existing LSs, and the conclusion is in Section 5.

## 2. Literature Review on Level Shifters

The LS [16] pulls out the static current via a PMOS cut-off. This LS is able to provide a strong inner node in comparison with other LSs, where the inner node is floating between voltages. To decrease energy consumption and delay within the design, the conflict between the “pull-up and pull-down networks” located within an inner node is decreased with a low-to-high transfer correction circuit, which degrades even at low frequency operations at near-threshold regions. The presented LS is designed with a 65 nm node. The results show that the lowest VDDL min is 0.12 V and 0.3 V with signal of 1 MHz, and 2 MHz at a higher VDDH of 1.2 V. The energy consumption and delay at a VDDL of 0.12 V are 1.04 p J and 207 ns, and for a VDDL of 0.3 V, the energy consumption and delay are31.25 fJ and 3.26 ns; the static power is 3 nW at a VDDL range of 0.12 V to 0.7 V.

In this brief [17], an accurate low-power and high-speed voltage LS is presented. With the use of a cross-coupled pull-up network, the power consumption is decreased and the switching speed is enhanced. The represented LS is competent in converting input signals with levels of voltage that are, to a great extent, lesser than the voltage at the MOS device cut-in voltage to the saturation levels. The represented LS covers a minute silicon area, allowing for, at the very least, no cores at the cost of the abnormal planner area.

The dual-output gate driver for switched-mode supplies of power can require lowest-side source signals and can be transformed to switch-node voltage. In the transfer to ultra-high switching GaN voltage converters, there are commercial requirement to gain switching node activities on slew rates increasing by 100 V/ns. Nevertheless, LSs do not work at the time to attain the necessary power supply at propagation delay in ns and slew immunities. This paper presents a four-level design methodology to design high-voltage slew rate protection by floating voltage LSs that meet the needs of GaN FET drivers. Slowly, transistor-level design methods are represented. These levels represent LSs for different areas, with the floating ground of532 ps and active energy of 30 pJ. It has a figure of merit of 0.06 ns/µmV and is nearly 1.7-times-developed at the complexity of the circuit design [18].

In this article, an area- and power-efficient LS is introduced. This LS is inferred from a replicate output Wilson-CMLS. The limitation of the Wilson-CMLS is in the incorporation of the CM; with a purely high sizing ratio, it can notably save the area and the power. The created LS works well with incredibly sub-threshold voltages of contribution, while displaying fundamentally low delay. Achieved in a 180 nm CMOS process, post-design models embracing the efficiency of LS can change the voltage of input levels as low as 0.05 V to around 1.8 V and occupy a silicon area of 7.5 × 4.7 µm^2^; the delay is 6 ns and the power consumption is 76.4 nW at 0.4 V/1 MHz input [19].

In this article, a robust LS structure can be capable to shift input from extreme sub-threshold voltage up to supply voltages. The addressed schematic is a self-biased low-voltage cascode CM technique that is attributed to diode connection. NMOS and PMOS are utilized to drive the input transforming inverter, which is utilized in the result stage with high energy effectiveness. This structure was implemented under standard 180 nm CMOS technology. The given circuit permits a voltage up-shift from 0.4 V to 1.8 V @ 100 KHz with an overall propagation delay of 7.6 ns at the typical energy transition, which is just 69 fJ at an area of 82 µm square [20].

In this article [21], an extremely low-voltage LS accompanied by implanted re-configurable logic is equipped for conveying two NAND and NOR logic activities with signals of input that are used to amplify signals of output to VDDH. The design is fabricated on a 45 nm node. It can convert the voltage of the input, which is 0.3 V, to the voltage of the output, which is 1.8 V, with the signal 1 MHz. The represented structure is extremely area-efficient at the input voltage of 0.3 V and output voltage of 1.8 V at 1 MHz. The overall propagation delay is 53 ns and power consumption is 35 nW.

The article [22] presents an improvement in the result of the HV floating LS. In these high-frequency gate drivers, for the most part, for large band gap uses, different voltages per ns noise result in high complexity. The delay of the achieved floating LS is radically decreased by using the edge detection method with supplementary pull-up circuits and endorsing propagation delay matching, and compatibility techniques are acquired. This LS is more appropriate for wide-frequency and wide-range applications.

In this article [23], an extremely low voltage LS accompanied by implanted re-configurable logic is equipped for conveying two NOR and NAND logic activities with signals of input which are used to amplify signals of output to VDDH. The design is fabricated in a CMOS 45 nm technology node. It can convert the input of 0.3 V to 1.8 V with a frequency of 1 MHz. The represented structure is extremely area-efficient and propagation delay is 53 ns and power consumption is 35 nW. 

The LSs in [24,25,26] designed and developed for sub nanoscale voltage conversions, capable of converting input voltage is greater than 0.30 V, the level shifted voltage is 1.8 V, and the input signal is 1 MHz. The overall propagation delay is appreciable and power consumption is low. The performance of the proposed structure is compared with the enlighten LSs [21,27,28,29].

## 3. Proposed Pass-Transistor-Enabled Split Input Voltage Level Shifter

This Pass-Transistor-Enabled Split Input Voltage Level Shifter (PVLS) is the modified version of the conventional LS, i.e., Wilson’s current mirror LS. The pass transistor logic facilitates the passing of strong logic 1 and weak logic 0, which reduces the leakage currents and signal coherence, with associated delays, depicted in Figure 4.

**Figure 5:**
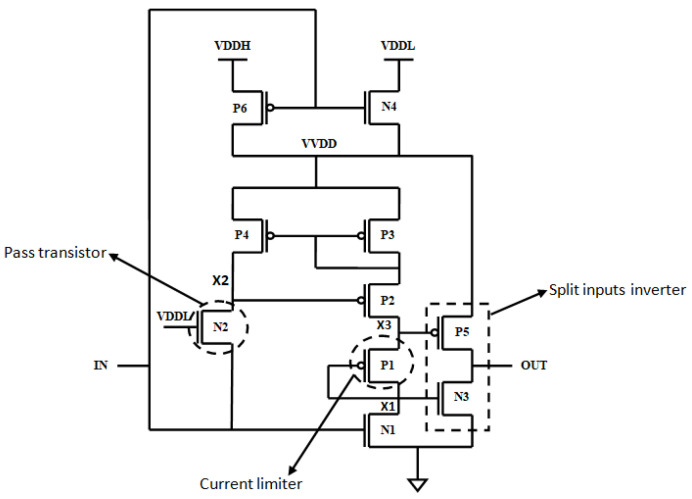
Pass-Transistor-Enabled Split Input Voltage Level Shifter.

The fundamental theory in the voltage transformation stage is the assimilation of one PMOS-Current limiter (P1) in the pull-up region to diminish current contention, depicted in Figure 5.

Multiple supply voltage results in a reduction in power and delayed improvement. The PVLS design is proven to be operated at sub-threshold regions to achieve ultra-low power and satisfactory performance metrics. The current Equations (1) and (2) symbolize the currents of *P*1 and *N*3 while operating in a sub-threshold area while at VDDL input:

(1) $${\;I}_{sub\;}=I_{0\;}e^{\frac{(V_{GSP1-}V_{T\;)}}{ɳV_{th}}}$$

(2) $${\;I}_{0}=\mu_{0\;}C_{ox}\frac{W}{L}\left(n-1\right)V_{thN3}^{2}$$

where *V_GS_*—potential across gate source, *V_T_*—threshold voltage, *V_th_*—thermal voltage, *µ*_0_—zero bias electron mobility, *n*—threshold factor of NMOS devices (1 + C_dep_*/C_ox_*), *C_ox_*—oxide capacitance of MOS devices, C_dep_—depletion capacitance of NMOS devices, *L*—length of gates, and *W*—width of gates.

The current limitation is a major exercise in analog circuit design and imposes a higher constraint that may be supplementary to a load to safeguard the LS turn-out or pass on glitch currents of a signal change over penalty because of a static circuit current within the LS for load based on Equation (3):

(3) $$I_{P1leakage}=\frac{W_{P1}}{W_{0P1}}I_{o\;}e^{\frac{\left(V_{gsP1}-V_{tp}\;\right)}{{ɳV}_{th}}}$$

(4) $$I_{DSN2}=\mu_{0}C_{ox}(\frac{W_{N2}}{L_{N2}})V_{T}^{2}\;exp\left(\frac{V_{gs}-V_{T}}{{\eta V}_{T}}\right)\left(1-\exp\left(\frac{-V_{DSN2}}{V_{TN2}}\right)\right)$$

(5) VOUTt=τGmP5CLVDDH−OUT−VthP5−τIN3CLVthN3etτ

where $V_{thP5}$ is the threshold voltage *P*5, *G_mP_*_5_ is the transconductance of the PMOS *P*5, $V_{DDH-OUT}$ is the voltage at output when *V_DDH_*, *V_thP_*_5_ is the titular *V_t_* of the PMOS *P*5, *C_L_* is the FO5 matchable capacitances of OUT, and *I_N_*_3_ represents current of the NMOS *N*3 transistor.

The represented design of this work is shown in Figure 4, including the voltage transformation stage and output buffer, i.e., split input inverter. The main modification at the voltage transformation stage is the PMOS-diode or transistor current limiter (P1), which is included in the pull-up side to exponentially diminish the current contention. Also, an additional circuit consisting of MOS device P6 and N4 is added to the LS, which will act as a 2 × 1 multiplexer; it will decide whether VDDL or VDDH supply is given to the circuit based on the given input. Suppose the input is high, then VDDL is given as supply for the LS circuit and, accordingly, the LS circuit gives the output as low; similarly, if the input is low, then VDDHis given as the supply for the LS circuit and, accordingly, the LS circuit gives the output as low. So, to avoid confusion, let us term it as a Virtual VDD (VVDD). In this way, it will decide the supply of the LS. Hence, the represented PVLS is capable of performing both level-up and level-down.

A PMOS-P1 is connected in series to NMOS-N1 to further decrease the power consumed by the circuit, and the driving buffer is a split input inverter, greatly reducing the short-circuit current by the output buffer. Let us assume that a low voltage is given as input; then, VDDH is given as the supply for the LS and it is termed as a VVDD. The necessary transistor-switching operation takes place and the corresponding output, i.e., high voltage, is obtained; similarly, if a high voltage is given as an input, then VDDL is given as the supply for the LS and it is termed as a VVDD. The necessary transistor-switching operation takes place and the corresponding output, i.e., the low voltage, is obtained.

When zero voltage is given as an input to the LS, then the output is also zero voltage; it does not perform any level shift. The PMOS transistor (P1) produces a voltage difference between X1 and X3 nodes to make sure that N3 and P5 donot turn on simultaneously in the output buffer. Additionally, as compared to Wilson’s current-mirror-based LS inverter, it is eliminated and changed by NMOS-N2 pass transistor inside the proposed structure. While the input is falling, the delay is reduced, which reduces the general propagation delay; subsequently, the circuit speed is eventually increased and, in addition, N1 and N2 perform inside the sub-threshold region, and are decided on as fingered ones to achieve a higher performance.

The PVLS design is capable of performing both level-up and level-down; the design is implemented under 55 nm technology using a cadence tool and, also, layout is extracted for the represented structure. In Table 1, depicts the aspect ratios, i.e., the W/L (nm) of PMOS from P1 to P6 and NMOS from N1 to N4. In a planarized manner and to give the best possible delay, power consumption and area below W/L values are considered.

## 4. Results and Comparison of PVLS

**Figure 6:**
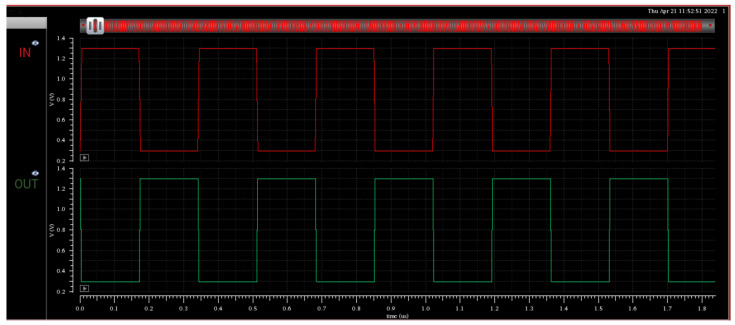
PVLS level-up/level-down @1 MHz.

The simulation waveforms of the represented voltage level shifter “Pass transistor Enabled Split Input Voltage Level Shifter” are depicted in Figure 6. It includes the waveforms of both level-up and level-down voltage conversion, ranging between 0.3 V and 1.3 V.

The proposed design is the takeover LS circuit; the leakage power consumed by the circuit is 2.90 nW and it offers a delay of 90 ps for an input frequency of 1 MHz. The developed LS is optimized in a 55 nm node and the simulation results are compared with the same node at various parameters in comparison with previous LSs, which are shown in Table 2. The PVLS could convert a minimum VDDL of 0.15 V to 1.30 V and vice versa at 27 °C and FO2. The number of transistors needed is 12 in the proposed PVLS, which have a highly efficient semiconductor design. The leakage power of the best design [29] is 2.32 nW, which is low with the convertible VDDH of 1.2 V. Comparatively to the other models that were tested, the proposed PVLS features a lower energy consumption and low delay. The delay of PVLS is far better than the best LS reported and useful as per [30].

Overall, the leakage power of the proposed PVLS is lower than [27]. However, the delay is drastically lower than [27,28,29]. Paper [1] described the material used for making the circuits; this material can be used for making an LS. The delay and power consumed by the proposed PVLS at various values of VDDL, VDDH at an input frequency of 1 MHZ, are analyzed. 

### 4.1. Layout of PVLS

**Figure 7:**
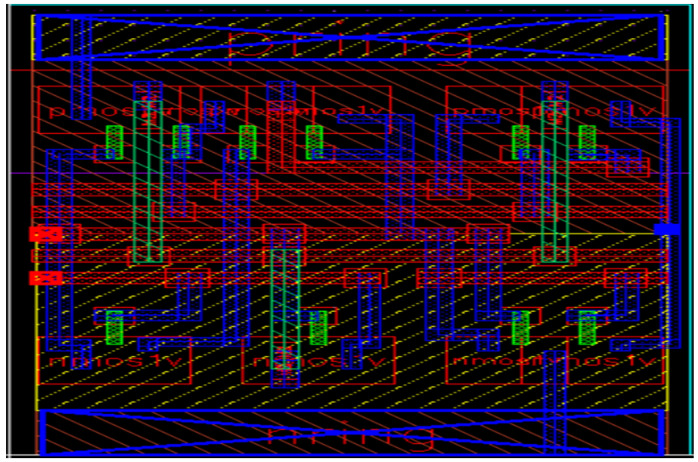
Layout of PVLS.

The physical layout is provided for the proposed PVLS and is also extracted after performing necessary validations of DRC and LVS checks to make sure that the functionality of the layout and schematic are perfectly matched, which is shown in Figure 7. The PVLS layout comprises n-wires, p-wires, and metal layers with the adaptive DRC and LVS having 2.90 μm × 2.64 μm with approximately 2.2 aspect ratios of NMOS and 3.0 aspect ratios of PMOS devices. It is obvious that the layout size of the MP5 transistor is the largest. Its size is lower in comparison with available LSs. The designed PVLS occupies approximately a 7.66 μm^2^ area.

### 4.2. Power and Delay of PVLS as a Function of VDDL

**Figure 8:**
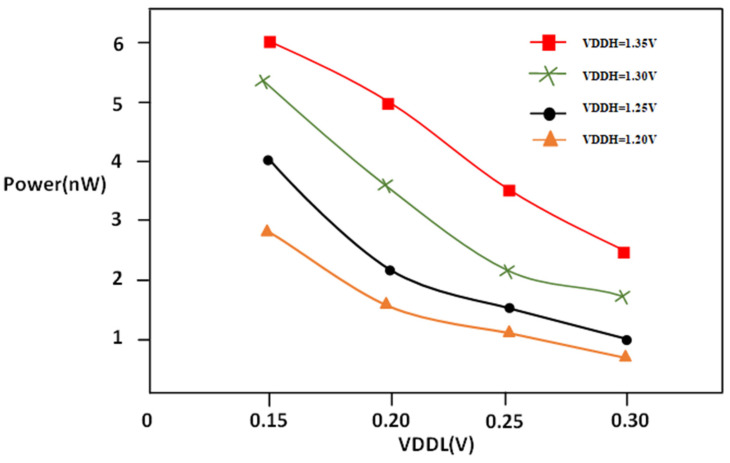
PVLS power vs. VDDL @1 MHz.

In Figure 8, it can be seen that supply voltage at a high level of LS can be affected by the energy consumption. In particular, power is directly related to VDDH, and delay of circuits is indirectly related to the percentage of the VDDH’s square. That means that an increase in VDDH results in an increase in the power consumed, which is quadratic. The high VDDH leads to higher power consumption; however, they can lower the time to propagation and enhance the efficiency of circuits. So, when deciding on VDDH levels for circuits, engineers need to strike the right compromise between reducing power consumption while maximizing efficiency. As VDDL increases, there will be a reduction in power due to switching the partially on transistor to fully on transistors in support of the multi-threshold CMOS technique and split inverter.

**Figure 9:**
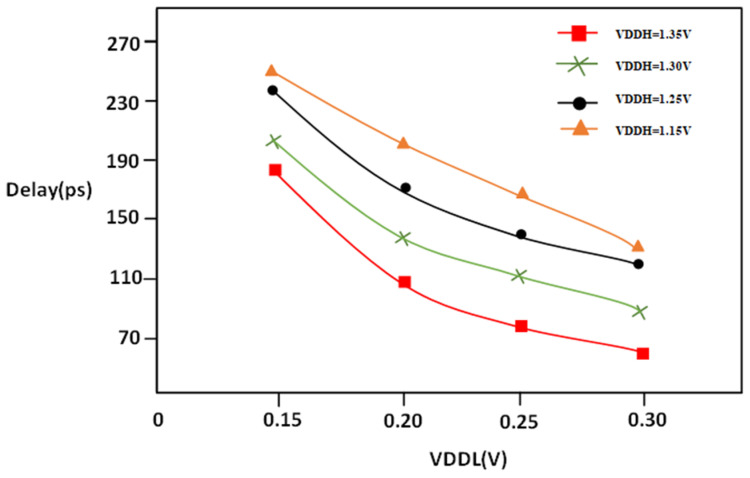
PVLS delay vs. VDDL @1 MHz.

The propagation delay measured against VDDL ranges between 0.15 V and 0.30 V with different values of VDDH, which are1.20, 1.25, 1.30, and 1.35 voltages. The supply voltage at the low level of digital circuits could affect the duration of the propagation delay. A similar equation to that of VDDH can be applied to clarify the relationship between the delay and power. The Equation (5) demonstrates how the delay is reducible when the VOUT node is influenced by transconductance; the one that is used for VDDH can be used to enable the connection between delay propagation, as shown in Figure 9.

### 4.3. Power and Delay of PVLS as a Function of VDDH

**Figure 10:**
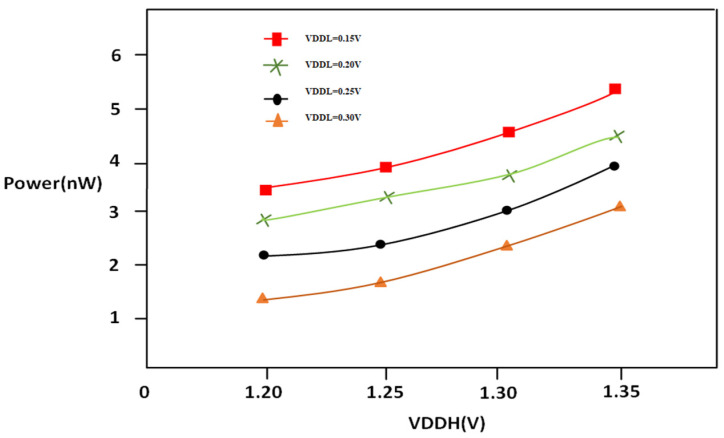
PVLS power vs. VDDH @1 MHz.

Referring to Figure 10, the power consumption is greatly affected by the I_DS_ of N2, as this device turning from non-saturation to saturation drags down the leakage and dynamic current, which lowers the power consumption at the highest level of VDDH. In the case of multi-threshold LS analog design, the power consumption is typically calculated as a quadratic value of VDDH. That is, when VDDH is increased, power consumption rises by a quadratic increase.

In other words, increasing VDDH leads to higher energy consumption, even though it could improve the performance of circuits by dropping the propagation delay; a load-balancing driving split inverter to limit high-static current creates low power consumption. This realization highlights the critical choice that engineers working on designing digital circuits have to make in order to preserve energy efficiency while attaining the required levels of performance. When it comes to the design and operation of the digital CMOS circuits, VDDH and power consumption are closely connected aspects.

**Figure 11:**
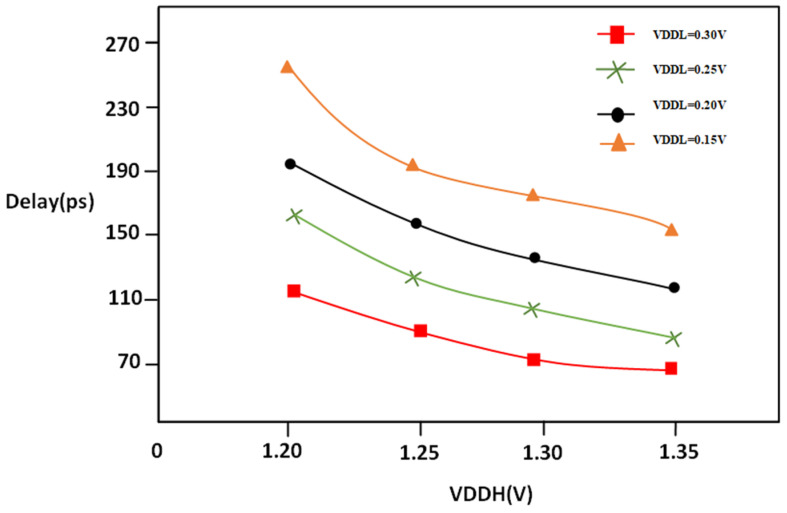
PVLS delay vs. VDDH @1 MHz.

The propagation delay measured in relation to VDDH can vary between 1.20 V and 1.35 V; with varied constant VDDL values, it ranges from 0.15 V to 0.30 V. There are a variety of variables that can affect the speed of propagation in digital circuits. One factor is the voltage of supply at high levels drastically reduces the delay, as exposed in Figure 11. The delay refers to the time that it takes the output of a digital circuit to alter in response to a change in its input. The length of time which passes between the moments of any change in the input signal can be replicated by the same shift in output, which is referred to as delay. In contrast, VDDH signifies the high-power supply voltage within the LS. It specifies the voltage that the LS is operating under when the level of logic 1 is on the output. The supply voltage and delay of design of VDDH generally have an inverted relation: the delay in propagation will decrease when the VDDH rises. From the simulation outcome, we may observe that, as the VDDL value increases power consumption, the delay decreases, and, if VDDH is increased, then power is increased and delay is reduced.

### 4.4. Power Delay Product of PVLS

**Figure 12:**
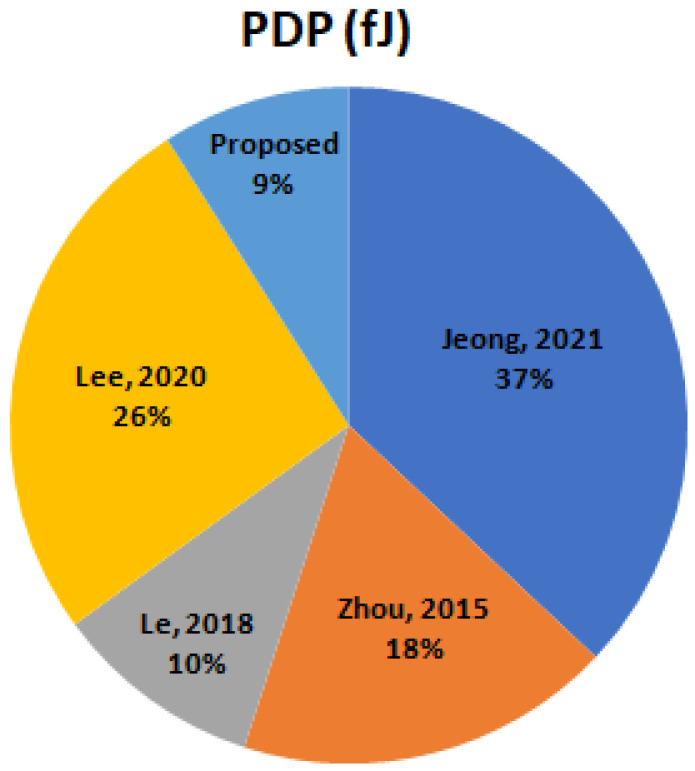
PDP of start art LS with proposed PVLS @1 MHz [23,24,25,26].

Figure 12 compares the power delay product of PVLS in a distinct perspective from the LSs [24,25,26]. In this example, VDDL is fixed to 0.3 V, whereas VDDH varies between 1.2 V and 1.35 V. With a fixed VDDL, the greater VDDH has two opposing impacts on speed and improved current ability: (1) the greater contention of LS due to a lower VDD slows down the speed and (2) the improved current ability of VDDH boosts the speed. Combining these two aspects make the time delay for LSs constant over VDDH.A notable exception is PVLS with a current limiter, with a significantly reduced delay. This is due to the fact that, for the virtual VDD that is generated, as shown in Figure 4, VVDD is added to the sub-threshold zone, which makes the initial-stage LS extremely high-speed. This is due to the fact that, at a higher VDDH, the delay of the chain inverter is not sufficient to ensure stability because of the threshold voltage adjustment, as mentioned previously.

## 5. Conclusions

A Pass-Transistor-Enabled Split Input Voltage Level Shifter is designed for area-, delay-, and power-efficient applications with a wide voltage conversion range. The represented low-power PVLS structure is a general blend of both pull-down and pull-up networks, which performs level-up or level-down shifts. The PVLS is incorporated with the multi-threshold CMOS technique and a load-balancing driving split inverter to limit high-static current, leakage power, and performance degradation. The results of the simulation were demonstrated using 55 nm process design software. The PVLS can convert a VDDL of 0.3 V to a VDDH of 1.3 V and vice versa. The proposed PVLS demonstrated a propagation delay of 90 ps and a leakage power of 2 nW with an area of 7.66 μm^2^ at an input signal of 1 MHz, showing great development in power efficiency in comparison with other LSs. Additionally, the PVLs have an isolation function as well as the power switch function. By using this design, it can cut down on extra cells to reduce the amount of area consumed, making this PVLS one of the most efficient designs. The suggested PVLS will result in moderate power consumption for dynamic applications due to the transition signals. Moreover, it is still aggressive, with the static power consumption much less than other designs, and, also, the delay is lowest among the best LSs. In real-world IoT applications, the standby power is more important because the IoT devices are usually in the sleep state.

## Figures and Tables

**Table 1:** Aspect ratio of the MOSFETs of the PVLS.

MOSFET	Aspect Ratio	MOSFET	Aspect Ratio
P1	3	P6	3
P2	3	N1	2.2
P3	3	N2	2.2
P4	3	N3	2.7
P5	3.5	N4	2.2

**Table 2:** Simulation results and comparison of PVLS with existing LS.

Ref./Proposed	Technology (nm)	Leakage Power (nW)	Delay (ns)
[27]	55	3.53	25.0
[28]	65	20.4	17.5
[29]	55	2.32	25.6
[21]	65	2.90	9.70
Proposed	55	2.90	90.0

## Data Availability

The data will be provided on the request basis.
